# Migration process of Venezuelan women to Brazil: living conditions and use of health services in Manaus and Boa Vista, 2018–2021

**DOI:** 10.1186/s12889-024-18109-5

**Published:** 2024-04-15

**Authors:** Maria do Carmo Leal, Thaiza Dutra Gomes de Carvalho, Yammê Ramos Portella Santos, Rita Suely Bacuri de Queiroz, Paula Andrea Morelli Fonseca, Celia Landmann Szwarcwald, Pía Riggirozzi

**Affiliations:** 1 National School of Public Health, Oswaldo Cruz Foundation, Leopoldo Bulhões Street, 1480, 8° floor, Bonsucesso, Rio de Janeiro, RJ 21041-210 Brazil; 2 Leônidas and Maria Deane Institute - Oswaldo Cruz Foundation - Fiocruz Amazônia, Street Terezina, 476 - Adrianópolis, Manaus, AM 69057-070 Brazil; 3 Institute of Scientific and Technological Communication and Information in Health, Oswaldo Cruz Foundation, Av. Brasil, 4365 - Manguinhos, Rio de Janeiro, RJ 21040-900 Brazil; 4 Department of Politics and International Relations, University of Southampton, Highfield Campus, Southampton, SO17 1BJ UK

**Keywords:** Brazil, Venezuela, Migrants, Social determinants of health, Women’s health

## Abstract

**Background:**

The last decade saw the emergence of a new significant migration corridor due to the mass migration of Venezuelans to neighboring countries in South America. Since 2018, Brazil became the third host country of Venezuelan displaced populations. Little is known about how migratory processes affect needs, access to social programs, and public health services of migrant women. The goal of this study is to shed light on the socio-economic profile, living conditions, and use of health services of Venezuelan migrant women in two main reception cities in Brazil.

**Methods:**

A survey was conducted using *respondent-driven sampling* (RDS) in the cities of Boa Vista (Roraima), and Manaus (Amazonas). The study included 2012 Venezuelan migrant women aged between 15 and 49 years old who migrated from Venezuela to Brazil between 2018 and 2021. Relative prevalence was calculated, and the χ^2^ test was used to analyse the homogeneity of proportions. All analyses considered the complex sampling.

**Results:**

The main reasons for migrating relate to difficulties obtaining food (54%) and accessing health services (37.8%) in their country of origin. They were young and mixed race (65.7%) and had high school education (69.9%). In Manaus, 3.7% of the interviewees declared that they had no family income in the last month, while in Boa Vista, it was higher (66.2%) (*p*-value < 0.001). Almost one-third of them sought health care in the last 15 days, and 95% of them received care. The residents of Boa Vista arrived more recently and family income and access to paid work improved with time of residence in Brazil.

**Conclusions:**

Given the increasing flow of Venezuelan migrants crossing to Brazil, a reception system was established for the provision of food, shelter, access to health services, and income transfer programs to migrants. This was the case despite high unemployment and poverty levels and income inequality, particularly in the city of Boa Vista. However, the majority had legal migrant status and had access to the public and universal healthcare system in Brazil (SUS). The use of the SUS was similar in both cities, acting as a buffer for the differences in opportunities offered.

## Background

According to the United Nations Refugee Agency (UNHCR) (2022), in recent decades, the number of forcibly displaced people has increased, with over 100 million people fleeing their home countries due to war, political persecution, and human rights violations [1]. In Latin America, more than 4 million Venezuelans left their country between 2015 and 2019, generating the largest migration exodus in the region’s history [2].

Venezuela has faced social, political and humanitarian crises concomitantly with a financial crisis, producing a 72% reduction in gross domestic product (GDP) between 2014 and 2020, all of which was also affected by the global oil crisis and embargoes imposed by Western countries. During this period, and in a context of hyperinflation and unemployment, Venezuela experienced negative population growth as women of reproductive age fled the country, deepening the birth deficit. Meanwhile, infant mortality rates also increased [3]. In 2021, 24.8% of the population experienced extreme poverty, 94.2% food insecurity, while 24.5% experienced severe food insecurity. The living standards deteriorated further as health services deteriorated rapidly, including a reduction in the number of prenatal consultations registered by the National Survey on Living Conditions – ENCOVI [4].

Faced with these adversities, millions of Venezuelans fled to neighbouring countries, in particular to Colombia, Peru, Chile, Ecuador and Brazil [2]. In Brazil, it is estimated that more than 700,000 people arrived between January 2017 and February 2022, including families with children and unaccompanied men and women [4].

Venezuelans crossed to Brazil mostly through Pacaraima, a small town in the state of Roraima with an estimated population in 2020 of approximately 20,000. Initially, Venezuelans were received by different local civil and religious societies as an expression of social solidarity and citizen mobilization. Soon, international organizations such as the International Organizations for Migration (IOM) and the UNHCR settled in the northern border of Brazil [5]. Boa Vista and Manaus, capitals of the states of Roraima and Amazonas, in the Amazon region, were the main destination cities for Venezuelan migrants in Brazil. Boa Vista and Manaus are challenged by poverty, lack of resources and unfunded social services. In 2019, out of the 27 states in Brazil, Roraima ranked at the very bottom while the state of ranked Amazonas 15th in terms of GPD. Not surprising, receiving such high flow of Venezuelan migrants, in a situation of great vulnerability, in such a short period, presented challenges for these cities that have already faced social inequalities for decades [6].

In this context, the federal government put in place a reception programme called Operação Acolhida, ran by the Brazilian Army and implemented with support of IOM and UNHCR. This was the first humanitarian operation on Brazilian soil that has operated on three fronts: border management and the reception of migrants; sheltering and healthcare provision; and internalization of Venezuelans to other cities in Brazil [7]. As a result, several temporary shelters were built in Roraima to house more than 6 thousand migrants in Pacaraima and Boa Vista [8]. Despite offering such social protection, housing and food, and helping in obtaining documents, migration management in shelters and in the approach to the provision of healthcare has been the target of criticism for privileging an overly security approach [5, 7].

In March 2020, the declaration of COVID-19 as a health emergency led to the closure of Brazil’s borders with Venezuela and consequently a reduction in the number of migrants arriving from Venezuela. However, far from deterring migration what the border closure produced was an increase of migrants crossing through irregular paths, called “trochas” [9]. Displaced women and girls, travelling by trocha is particularly marked by challenges of health, particularly sexual and reproductive health, due to increased risk of for violence, lack of personal and menstrual hygiene, inadequate access to safe, clean, and private toilets, and exposure to sexual violence [10–12].

We situate our analysis and the discussion of results in the remaining sections, in this context, noting that in the period 2018–2022, Brazil was governed by an elected conservative, far-right government that reduced resources for social programs related to health, education, housing and income transfers, and did not adjust wages for inflation, which, together with the COVID-19 pandemic, led to high levels of unemployment (14.9% for men and 18.5% for women) and poverty (29.4%) and a return to food scarcity (hunger) in Brazil [13, 14].

The analysis that follows is based on data from a survey on the sexual and reproductive health of Venezuelan migrant women in Brazil, as part of the “Redressing Gendered Health Inequalities of Displaced Women and Girls in Contexts of Protracted Crisis in Central and South America – (ReGHID)” project, coordinated by the University of Southampton and funded by the Economic and Social Research Council of the United Kingdom, were analysed. The aim of this study was to assess the migration process of Venezuelan women of reproductive age, their socioeconomic characteristics, and their access to and use of health services in the cities of Boa Vista (RR) and Manaus (AM), northern Brazil, between 2018 and 2021.

## Methods

### Sample size

To calculate the sample size, the reference parameter was the use of health services by migrant women in the 15 days prior to the survey. In Brazil, according to data from the 2019 National Health Survey, the percentage of people who received health-related care in the 2 weeks prior to the survey was 18.6% [15]. Therefore, the minimum sample size was calculated to estimate a percentage of 19% with a two-sided error of 4%, considering a design effect of 2. The estimated sample size was 730 women for each city.

The survey was conducted in the cities of Boa Vista, capital of the state of Roraima (RR), border with Venezuela and main city of arrival, and in Manaus, capital and largest city in the State of Amazonas (AM) and the second host city for Venezuelan migrants. Respondent-driven sampling (RDS) was used [16] as a strategy to identify migrant women residing in the two Brazilian capitals. RDS is an appropriate methodology for populations that are difficult to access when the actual size or location of populations to be accessed is not known. RDS is performed through successive recruitment cycles. This method allows calculating the probability of selection through the size of the network of each participant [17]. Taking into account the connections among the participants, the sample can be considered a cluster sample with different probabilities of selection [18].

We started with what is called ‘seeds’, Venezuelan women selected to start the chain recruitment process. After participating in the study, each seed received three invitations (coupons) with unique codes generated by the invitation manager to invite other women from the same target population. This process was repeated until the sample size in each city was reached.

To manage the invitations, a Microsoft Access program was developed that included the eligibility form and a unique code assigned to each participant. The code made it possible to identify all recruiter-recruited pairs, allowing the estimation of the effects of homophily, which occur because participants have a tendency to invite others with characteristics similar to theirs [19] .

The eligibility criteria were as follows: (i) women, (ii) Venezuelan, (iii) between 15 and 49 years of age, (iv) in Brazil for a maximum of 3 years, (v) a valid invitation to participate in the study, and (vi) had not previously participated in the study. The 3-year criterion was used to avoid memory bias regarding the migratory path and simultaneously study the short-term impact of length of residence on the living and health conditions of these women in Brazil.

Each interviewee received financial support for the costs of transportation and food on the day of the interview and a secondary incentive for each participant they recruited who completed the questionnaire.

In the first stage of the study, the women were interviewed by field supervisors who completed the eligibility form and validated the invitation with a barcode. At that time, the women were also asked about the size of their network using two questions: “How many Venezuelan women who have moved to Brazil in the last 3 years do you know personally, and do you know their names?” and “How many of these women are between 15 and 49 years of age?”. Information pertaining to the recruitment pairs and the size of the networks of each participant were used to adjust for complex sampling in the calculation of sample weights and clusters.

Data collection was performed through structured interviews conducted by Venezuelan interviewers trained by the Fundação Oswaldo Cruz - Fiocruz team to administer the questionnaire, which was developed specifically for this study. The questionnaire included sociodemographic questions about the migration process, work and income, spending on health, habits and lifestyles, use of health services, women’s health, reproductive history, prenatal care, childbirth, abortion and COVID-19 occurrence, immunization and hospitalization. A questionnaire about housing characteristics was also applied. All interviews were conducted in Spanish. The data were collected using RedCap - Research Electronic Data Capture electronic data capture tools hosted at Oswaldo Cruz Foundation [20, 21].

The interviews took place in 2021, between July and August in Manaus and in September in Boa Vista; 761 and 1267 migrants, respectively, participated. After cleaning the database and excluding the seeds, the data of a total of 2012 interviews were analysed.

Relative prevalence was calculated with 95% confidence intervals. The χ^2^ test was used to analyse the homogeneity of the proportions. The analysis was conducted separately for the two cities and subsequently stratified by length of residence in Brazil.

The migrants were analysed by place of residence, Manaus and Boa Vista, to isolate the effect of the differences in economic and social opportunities offered by the two cities and substantial difference in shelter and support offered through Operação Acolhida.

To calculate the prenatal adequacy, a minimum of six appointments recommended by the Brazilian Ministry of Health for low-risk pregnancies was considered, i.e., at least one appointment in the first trimester, two appointments in the second trimester, and three appointments in the last trimester [22].

All analyses considered complex sampling and were conducted using SPSS software (IBM SPSS Statistics for Windows, Version 23.0. Armonk, NY: IBM Corp).

The project was approved by the Research Ethics Committee of the Federal University of Maranhão under number 35617020.9.1001.5087.

## Results

As seen in Table 1, the age distribution of Venezuelan migrants in the cities of Boa Vista and Manaus was similar, where most migrants (35.8%) were between the ages of 25 and 34 years. Regarding education, in Manaus, 21.2% of the migrants had completed higher education, while in Boa Vista, only 10.3% completed that level (*p*-value < 0.001). Among the study population 65.7% self-reported as mixed race, 28.2% self-reported as white, and 2.3% self-reported as indigenous. There were more indigenous and black women in Boa Vista than in Manaus (*p* value < 0.001). In both cities (61.3%), the majority declared that they had a partner. In Manaus, 3.7% of the women reported that they had no family income in the last month, and in Boa Vista, 66.2% of the women reported the same (*p* value < 0.001 (Table 1).

**Table 1:** Sociodemographic characteristics of Venezuelan migrants that arrived in Manaus and Boa Vista between 2018 and 2021

Socioeconomic characteristics	Manaus	Boa Vista	Total	*P*-value
	*N* = 755	*N* = 1257	*N* = 2012	
	%	%	%	Est.
**Age**				0.629
15 to 19 years	13.2	15.1	14.4	
20 to 24 years	23.5	23.0	23.2	
25 to 34 years	35.1	36.2	35.8	
35 to 49 years	28.1	25.7	26.6	
**Education**				< 0.001
> = Elementary School	12.2	17.9	15.8	
High school	66.5	71.8	69.9	
<= University education	21.2	10.3	14.4	
**Race/color**				< 0.001
White	29,0	27.7	28.2	
Brown	67.9	64.4	65.7	
Black	1.4	4.3	3.2	
Indigenous	0.8	3.3	2.3	
Other	1.0	0.3	0.6	
**Marital status in Brazil**				0.676
Don’t have a partner	39.8	38.1	38.7	
Have a partner	60.2	61.9	61.3	
**Family income in the last month**				< 0.001
No income	3.7	66.2	43.5	
1 to 106 USD	16.8	17.3	17.1	
106 to 213 USD	52,0	12.0	26.5	
Above 213 USD	27.5	4.5	12.8	

For many the migration process involved paying smugglers to facilitate the crossing. In this study 28.4% of the Venezuelan women living in Manaus and 44.3% of the women living in Boa Vista paid someone who promised them migratory facilities (*p* value < 0.001). Most women were accompanied (87.5%), but a higher percentage of those who lived in Manaus migrated alone (17.9%, *p* value < 0.001). Pregnancy and motherhood are two distinctive gendered features amongst the study population. Of this, a higher percentage of women in Boa Vista reported having migrated pregnant (9.2%, *p* value < 0.018), and there was no difference between the two cities with regard to the percentage of women who left children in Venezuela (26.6%). Brazil was the final migration destination for most women (93.1%), with most (69.3%) reporting that they wanted to return to their country of origin.

The main declared reasons given by women for migrating were difficulty obtaining food (54.0%); difficulty accessing health care (37.8%); violence and insecurity (27.3%); and difficulty finding a job (23.2%), with higher percentages in Manaus (Table 2).

**Table 2:** Migration process of Venezuelan women that arrived in Manaus and Boa Vista between 2018 and 2021

Migration Process	Manaus	Boa Vista	Total	*P*-value
	*N* = 755	*N* = 1257	*N* = 2012	
	%	%	%	Est.
**Worked before migrating to Brazil**	69.7	55.1	60.6	< 0.001
**In this work, there was a formal bond**	42.5	21.5	30.6	< 0.001
**Main reasons for migrating to Brazil**				
Looking for or got a job	27.5	20.6	23.2	0.004
Study reasons	9.1	17.2	14.2	< 0.001
Difficulties accessing health care	37.2	38.1	37.8	0.762
Violence and insecurity	31.1	25,0	27.3	0.017
Difficulty getting food	59.3	50.8	54.0	0.002
Family reunification	9.6	9.7	9.6	0.983
**Paid someone who promised him facilities to enter Brazil**	28.4	44.3	38.3	< 0.001
**With whom did you migrate**				< 0.001
Alone	17.9	9.3	12.5	
Accompanied	82.1	90.7	87.5	
**Arrived pregnant in Brazil**	5.7	9.2	7.9	0.018
**Left children in Venezuela**	24.6	27.8	26.6	0.187
**In which country will you complete your trip**				0.013
Brazil	85.4	94.3	93.1	
Other country	14.6	3.3	4.5	
Do not Know	0.0	2.4	2.1	
**Want to return to Venezuela**				< 0.001
No	16.1	20.8	19.0	
Yes	77.1	64.6	69.3	
Do not Know	6.8	14.7	11.7	

Upon arriving in Brazil, 45.5% of the migrant women had support or help from the Brazilian government to obtain documents. Ninety-one percent of the women obtained an Individual Taxpayer Registry (*Cadastro de Pessoa Física-CPF*), 46.5% of the women in Manaus and 87.5% of the women in Boa Vista obtained a National Health Card (NHC) (*p* value < 0.001) (Table 3).

**Table 3:** Living conditions of Venezuelan women who arrived in Manaus and Boa Vista between 2018 and 2021

Living conditions in Brazil	Manaus	Boa Vista	Total	*P*-value
	*N* = 755	*N* = 1257	*N* = 2012	
	%	%	%	Est.
**Received some support or help obtaining documents or work**	53.0	58.4	56.4	0.062
**From who?**				
Operation Acolhida/ (PITRIG)	43.2	46.9	45.5	0.195
NGOS (International Fraternity, Fraternity Without Borders, AVSI, ADRA, CICV, MSF)	2.0	8.2	5.9	< 0.001
International organizations (ACNUR, IOM, UNICEF, etc)	3.9	10.0	7.7	< 0.001
**Documents obtained in Brazil**				
CPF	91.6	90.6	91,0	0.567
NHC	46.5	87.5	72.1	< 0.001
Work card	18.1	25.0	22.4	0.003
**Migratory status**				< 0.001
Asylum seeker	29.4	51.3	42.9	
Temporary/permanent resident	44.6	43.3	43.8	
Irregular	26,0	5.4	13.2	
**Paid work in the last month**	45.2	11.8	24.3	< 0.001
**Formal work**	4,0	6.9	5.1	0.016
**Received financial aid from the Brazilian government**	38.2	8.6	19.8	< 0.001
**Type of residence**				< 0.001
Shelter	0.6	68,0	42.7	
Collective housing	9.6	8.6	9,0	
Detached house	73.6	19.1	39.5	
Homeless	0.0	3.5	2.2	
Other	16.3	0.7	6.5	
**Send financial help to a family member in Venezuela**	65.1	21.7	38.0	< 0.001

Regarding migratory status, 29.4% in Manaus and 51.3% in Boa Vista were asylum seekers (*p* value < 0.001). A total of 43.8% in both cities were temporary or permanent residents. Twenty-six percent of the migrant women in Manaus and 5.4% in Boa Vista (*p* value < 0.001) had an irregular migration status (Table 3).

Almost half of the Venezuelan women in Manaus had worked for pay in the last month, and 11.8% of those in Boa Vista reported the same (*p* value < 0.001); however, only 5.1% had formal work in Brazil. In Manaus and Boa Vista, 38.3 and 8.6% of women, respectively, received financial resources through cash transfer programs (*p* value < 0.001). There were also differences in the type of housing; in Manaus, 73.6% of the women lived in individual residences, while and in Boa Vista, 68% lived in shelters, with 3.5% reporting not having housing at all (*p* value < 0.001) (Table 3).

Regarding the use of health services in Brazil, 31.7% of the women reported seeking health care in the last 2 weeks, and 95.0% received care for various reasons: illness (23.3%); prevention, periodic health care or childcare (19.4%); and vaccination (16.8%). Among the pregnant women and those who had children in the last 12 months in Brazil, 85% reported having received prenatal care, with large differences between cities when assessing the adequacy of such care: 81.2% for women in Manaus and 45% for women in Boa Vista (*p* value < 0.001) (Table 4).

**Table 4:** Access to health services in Brazil by Venezuelan women who arrived in Manaus and Boa Vista between 2018 and 2021

Access to health services in Brazil	Manaus	Boa Vista	Total	*P*-value
	*N* = 755	*N* = 1257	*N* = 2012	
	%	%	%	Est.
**Health service use in the last 2 weeks**	31.1	32.0	31.7	0.715
**Reason for using the health service**				0.326
illness	20.1	25.1	23.3	
Diagnostic test (blood, urine, etc.)	8.0	7.6	7.8	
Vaccination	19.5	15.2	16.8	
Prevention, check up or childcare	22.5	17.6	19.4	
Others	17.0	21.2	19.7	
* For women who are pregnant at the time of the interview*				
Prenatal care	85.8	85.3	85.5	0.121
**Adequacy of prenatal care adjusted for gestational age**^a^	81.2	45.0	62.0	< 0.001

^a^Only for women who were pregnant or had a child in the last 12 months in Brazil. It was considered adequate when there was at least 1 consultation in the first trimester of pregnancy, two consultations in the second trimester and three for those who reported being in the third trimester of pregnancy. And at least six consultations for those who gave birth in Brazil in the last 12 months

The time of arrival in Brazil varied between the cities. In Manaus,14.7% of the migrant women arrived within 6 months, and 61.5% in Boa Vista (Table 5).

**Table 5:** Socioeconomic characteristics according to time of residence in Manaus and Boa Vista between 2018 and 2021

Time of residence in Brasil	Manaus				Boa Vista			
	Up to 6 months	Between 6 months and 1.5 years	Over 1.5 year	*P*-value	Up to 6 months	Between 6 months and 1.5 years	Over 1.5 year	*P*-value
	%	%	%	Est.	%	%	%	Est.
**Education**				0.997				0.092
<=Elementary school	13.5	10.1	13.1		17.0	16.5	23.4	
High school	67.9	67.2	65.8		74.2	71.6	62.7	
> = University education	18.6	22.7	21.1		8.7	12.0	13.9	
**Family income in the last month**				0.017				< 0.001
No income	8.2	5.3	1.7		76.7	61.7	31.4	
1 to 106 USD	19.9	20.2	14.3		14,8	17.5	26.5	
106 to 213 USD	41.1	49.1	56.3		6.6	16.1	27.7	
Above 213 USD	30.9	25.5	27.7		1.9	4.7	14.4	
**Paid work in the last month**	35.5	39.6	50.9	0.008	7.2	15.1	24.9	< 0,001
**Migratory status**				< 0.001				< 0.001
Asylum seeker	14.2	25.9	35.0		54.8	52.2	36.8	
Temporary/permanent resident	16.6	24.9	62.4		38.1	43.1	63.2	
Irregular	69.2	49.3	2.6		7.1	4.7	0.0	

## Discussion

The Venezuelan migrant women who lived in Boa Vista and Manaus were young and predominantly mixed-race and white, had a high school education and low level of income and employment, and lived with a partner. Those residing in Manaus had a higher level of education, had more paid work or financial aid from the Brazilian government, and lived in individual households. In Boa Vista, the migrants lived mainly in shelters and had no family income. The main motivations for migrating to Brazil were difficulty obtaining food and accessing health services. Migrants residing in Boa Vista arrived more recently than did those in Manaus, and education, family income, access to paid work and regularization of migratory status improved with time of residence in Brazil.

As Venezuelans mostly migrated and remained in the northernmost states of Brazil, Roraima and Amazonas, closest to the Brazil-Venezuela border, a local socio-economic crisis began due to the unprecedented population increase. This was particularly the case in Roraima, one of Brazil’s poorest states, with little resources to respond to the increasing pressures on local services, where 10% of the population was estimated to be Venezuelan migrants by December 2018 [23]. To a large extent, the choice of Boa Vista and Manaus as places of residence is strategic due to their proximity to Venezuela, thus reducing the cost of sending goods to family members, allowing greater contact with family members through travel at low cost, and making it easier to bring older family members (parents) and children for family reunification [24, 25]. Most interviewees come from the districts of Bolívar, Anzoátegui, and Monaguas, which are the closest to Brazil. These states have high levels of poverty (above 95% of the population), like most states in Venezuela [3] .

At the time of the COVID-19 health emergency in 2020, with the closure of the border, the illegal market for crossing at “trochas” expanded into indigenous territories, which are not monitored by the Brazilian federal police and army. A significant portion of the women (38.3%), with a predominance among those interviewed in Boa Vista, had to pay someone who promised to facilitate the crossing, increasing the vulnerability of these migrant women to situations of extortion, abuse and violence [9, 26].

Those who migrated between 2018 and 2021 had a better level of education in the first year than in the last year, evidencing continued impoverishment upon the arrival of these groups, similar to the ENCOVI 2021 data [3]. The income of female migrants, although very low, improves with the length of stay in Brazil. Compared with that in Manaus, where professionally qualified women arrived earlier, the situation in Boa Vista is worse. In addition, Manaus offers greater opportunities for work and income.

The presence of irregular migrants was higher in Manaus than in Boa Vista probably because of the greater ease of regularization in the latter, where most migrants live in shelters built through Operação Acolhida. In 2019, Brazil classified Venezuela as a country with serious and widespread human rights violations, allowing Venezuelans to be considered refugees [27]. For this reason, in Manaus, where women have been in Brazil for longer, there is a greater frequency of permanent and temporary residents, and in Boa Vista, there is higher percentage of asylum seekers.

Having documents in Brazil does not guarantee entry into the labour market, as only 12% in Boa Vista and 45% in Manaus reported paid work in the month prior to the interview, with most performing informal work activities and having precarious contracts. These data reinforce the need for intersectoral policies, given that employment and income opportunities are essential factors for the integration and socialization of migrants in a host country [28]. The UN recommends that governments should assume their share of responsibility and commitment in coordinating efforts with nongovernmental organizations and civil society, adjust and align short- and long-term strategies that respond to the consequences of migration and meet the social needs and rights of migrants [1]. However, this is a very challenging issue to address in South–South migration, where receiving countries, as in the case of Brazil, are profoundly unequal and have high rates of unemployment and poverty among their own populations [14].

In Brazil, any Brazilian or foreign person living in poverty or extreme poverty can request benefits from an income transfer program such as Bolsa Família. Notably, a significant portion of women received some type of financial aid from social programs in Brazil, with the percentage being higher in Manaus than in Boa Vista [29]. This most likely occurred because those who lived in Boa Vista had less access to information to request the benefits and even those who have already requested have not yet had the benefit released because they been in the country for less time than those in Manaus.

The lack of food in their country of origin was the main reason cited by the women for migrating. As described above, hunger and food insecurity in Venezuela were identified by ENCOVI and Brazilian researchers [3, 30]. These challenges affect women more intensely because they are the main individuals responsible for the care of children. This study did not evaluate food insecurity and adequate access to food, as almost all women in Boa Vista had access to three meals offered by Operação Acolhida and local religious and civil society organizations [5]. Although the guarantee of housing and food alleviates basic needs, it does not eliminate the social vulnerabilities to which these women and their children are subjected. Extreme poverty, associated with the total absence of income for most of them, is superimposed on gender and migrant discrimination. Communication difficulties, due to both language and cultural differences, have led some authors to evoke the concept of structural vulnerability for migrants. This concept refers to two main components of capitalist societies: an economy based on class, culture, gender and race discrimination, symbolic processes of violence and the formation of subjectivities that legitimize punitive discourses for marginalized populations, such as migrant populations [31]. There are reports of harassment, attempts at expulsion, protests against the entry and stay of Venezuelans in Brazil, aggression, labour and sexual exploitation and unfounded accusations of crimes not committed [5]. The exacerbated vulnerability to which migrants are subjected disproportionately affects their health, not because they have worse health conditions than the host population but because of the effects of the interaction between social determinants of health, such as education, income, housing and the barriers of culture and language to which they are subjected [32]. Although most of the women in this study migrated accompanied by family members, one-quarter of them left one or more children in Venezuela, breaking their family ties, which contributes to increasing the psychological suffering inherent to the condition of migrants and the occurrence of mental disorders [33, 34]. A systematic review concluded that despite the diversity of situations, there was a consistent association between the separation of migrants from their families and impairments in mental health, ranging from well-being - psychological being to feelings of guilt, anguish, anxiety, and depression and posttraumatic disorders [35]. Other studies that have focused on migrant women who left their children suggest that depressive symptoms and emotional distress are prevalent among such women [36].

In Brazil, because the right to health is a universal and constitutional right for all, national and non nationals have the right to access to the SUS, and hence all health services are available for migrants regardless of their legal status and documentation [37, 38]. However, obtaining the National Health Card, often provided as part of the Operation Welcome/ Operação Acolhida, is needed for the effective access to healthcare facilities and services [39] .Given that the right to health is safeguarded by Constitution as a right for all, and given the deterioration of health conditions and services in Venezuela since 2014, it is likely that access to health services explain why this was the second reason given by the Venezuelan women for fleeing to Brazil; a fact that becomes more significant considering the high number of women who migrated pregnant. Yet, migration between countries in the global South generates demands on already precarious social and health systems that are rarely adequately equipped to respond to the internal needs of their populations, with a lack of infrastructure, personnel and equipment. COVID-19 pandemic put further pressure on health systems like SUS to in a timely and appropriate manner. However, despite the unfavourable scenario, the access to and use of health services by Venezuelan migrants was prevalent and diversified, probably reflecting both repressed demand and health needs arising from the effects of migration. Although the effectiveness and quality of care provided was not assessed in this study, when comparing the use of the SUS by the Brazilian population, that by migrant women was almost three times greater than that reported for residents of the North region in the 2019 National Health Survey [15]. Communication barriers due to cultural and linguistic differences between migrants and health professionals were identified by the qualitative study of this project [26], which was also verified with Bolivian migrants in other Brazilian cities [39, 40]. The differences in sociodemographic, housing and migratory status conditions between Boa Vista and Manaus were not noted as factors affecting access to health services. The SUS acted as a protective barrier against social inequities. The greater inadequacy of prenatal care observed for women in Boa Vista can be explained by the number of women who arrive pregnant to that city and therefore unable to complete the recommended number of appointments. Regarding satisfaction with the care received, a study of Venezuelan migrants living in shelters in the state of Roraima found that 75% of women declared themselves satisfied or partially satisfied with the sexual and reproductive health services received at health units in Brazil [41]. However, the prenatal care indicators for migrants were lower than those observed for Brazilian women in the North region [42].

This study has limitations. Among them, the recruitment of participants through RDS may have increased the representation of women who lived in shelters or in collective housing in this sample due to the greater ease of communication.

As positive points, the epidemiological data allowed working with average estimates for the target population. In addition, the survey included a representative sample that reflects the population of Venezuelan women who migrated to Brazil between 2018 and 2021 and how they are received in the country, including access to the health care system. The sample was large enough to analyse the study data in a population with difficult access. The interviewees were recruited by Venezuelan women, also migrants, thus facilitating communication and willingness to participate in the study.

The literature on migration and health has reported how women, adolescents and children are the most vulnerable groups in the migratory process, particularly if this is forced migration [43, 44], although these studies have mainly been concentrated on the experience of migratory flows to Europe [11]. The experiences of those in the move and the impacts on health, particularly of women in forced displacement within South-South migration is still under-researched.

Our study contributes to these concerns in two ways, bringing a new geographical focus that not only highlights characteristic so migrant women within southern displacement corridors, but also a focus on cities and the socio-economic and contextual determinants are important aspects that affect health and healthcare access and utilisation of services within groups of migrants in cities of settlement. The study allows identification of social determinants of health, in the understanding that any action on health equity should be through action on the social determinants of health.

Furthermore, while research on inequities in health and access to healthcare of migrants and refugees has focused on disparities between refugees and resident populations, this study offers new data on different determinants of health across cities in a region of settlement.

## Conclusions

One characteristic aspect within South-South migration, is that often displaced populations meet saturated local service provision and labour markets, uneven development and austerity, particularly in (border) areas. The Venezuelan women who left their country due to essential needs such as food, health care, and safety to live in the cities of Manaus and Boa Vista, in Brazil, they had low wages and precarious jobs and lived in shelters, without privacy, but also had a legal migrant status. Their situation was more precarious in Boa Vista than in Manaus, still, there was no difference in access to health services between the two cities, except for the adequacy of prenatal care. They used health services three times more than Brazilian women. There is a need to train health professionals to reduce communication and cultural barriers, seeking to recognize the specific health needs of migrants. Despite Brazil being a country in the global south with high indicators of unemployment and income inequality, it provided food, shelter, access to health services, and income transfer programs to migrant women who lived in Manaus and Boa Vista. While there is an emphasis on reception, sheltering and healthcare provision, it is still to be seen whether those commitments turn into long-term public policies supported by funding necessary to respond beyond humanitarian emergencies towards durable solutions. As it stands, the fact that most migrants in border areas like Roraima still have to keep moving towards other cities that may be in better socio-economic conditions, risk creating protracted displacement and failing to redress social and health inequalities that affect not only populations in displacement but those left behind for reasons of uneven, unequal and neglectful development.

## Data Availability

The datasets analyzed during the current study are available from the corresponding author at https://arcadados.fiocruz.br/dataset.xhtml?persistentId=doi:10.35078/3B7S4N.

## Abbreviations

AM: Amazonas
CPF: Cadastro de pessoa física
ENCOVI: Encuesta nacional de condiciones de vida
GDP: Gross domestic product
IOM: International organization for migration
NHC: National health card
RDS: Respondent driven sampling
REGHID: Redressing gendered health inequalities of displaced women and girls in contexts of protracted crisis in central and south america
RR: Roraima
SUS: Sistema único de saúde
UN: United nations
UNHCR: United nations high commissioner for refugees
